# Management of preoperative hypertension and anxiety based on early monitoring of pulse rate before cataract surgery

**DOI:** 10.1007/s10384-024-01124-9

**Published:** 2024-09-28

**Authors:** Takashi Ono, Takuya Iwasaki, Rie Tomari, Toshihiro Sakisaka, Yosai Mori, Ryohei Nejima, Kazunori Miyata

**Affiliations:** 1 Department of Ophthalmology, Miyata Eye Hospital, 6-3 Kuraharacho, Miyakonojo, Miyazaki 885-0051 Japan; 2 Department of Ophthalmology, Graduate School of Medicine, University of Tokyo, Tokyo, Japan

**Keywords:** Cataract, Heart rate, Ophthalmic surgery, Perioperative hypertension, Pulse

## Abstract

**Purpose:**

Few studies have addressed the clinical impact of fluctuation in pulse rate before cataract surgery. This study aimed to determine the effectiveness of prior pulse monitoring and intervention to prevent blood pressure changes in patients undergoing cataract surgery under local anesthesia.

**Study design:**

Retrospective study

**Methods:**

Patients who underwent cataract surgery under local anesthesia were included. In the pulse rate (PR) group, intervention was performed on the basis of early monitoring of blood pressure and PR changes. In the conventional group, blood pressure was managed on the basis of blood pressure alone. Systolic blood pressure, diastolic blood pressure, intraoperative nicardipine use, and physician consultation time were retrospectively compared between the groups.

**Results:**

The study included 684 eyes from 684 patients aged 73.5 ± 9.5 years (PR group: 297 eyes, conventional group: 387 eyes). No intergroup differences were found in systolic blood pressure or pulse and heart rates; however, diastolic blood pressure was significantly lower in the PR group than in the conventional group at both the beginning and the end of surgery (*P* <.001 and *P* = .0028, respectively). Intravenous nicardipine administration in the operating room was significantly less frequent in the PR group (*P* = .041), and physician consultation time after entering the operating room and at the beginning of surgery was significantly shorter in the PR group (both *P* <.001).

**Conclusion:**

Early monitoring of PR with blood pressure and intervention were effective for preventing preoperative hypertension.

## Introduction

Cataract surgery is often performed under local anesthesia because it is completed within a short time and the physical invasiveness is minimal. However, many patients are anxious about eye surgery under conscious conditions [1]. It is well known that anxiety can cause changes in vital signs, and Boker and colleagues reported that 60% of patients scheduled for elective surgery are known to experience anxiety [2]. Preoperative anxiety requires higher doses of anesthetic drugs [3, 4] and increases catecholamine secretion, causing tachycardia, hypertension, and arrhythmia [5, 6]. This perioperative increase in blood pressure has been observed during cataract surgery regardless of the host’s underlying disease (eg, metabolic syndrome [7]), suggesting that patients’ anxiety about the operation may contribute more to the increase in blood pressure. While benzodiazepine (BZD) prescribed for anxiety has anticholinergic properties and increases the risk of acute angle-closure glaucoma [8–10], inducing agitation as a paradoxical reaction is also a risk [11]. Given these risks, BZD cannot be uniformly administered as premedication to patients undergoing anterior cataract surgery.

However, a sudden increase in blood pressure carries the risk of cardiovascular failure. Abdominal aortic aneurysms are particularly likely to be found in older adults, with a reported prevalence of 1–7% (although there are racial differences) among patients aged ≥65 years [12], ≥64 years [13], and ≥60 years [14]. Additionally, older patients undergoing cataract surgery may have undetected abdominal aortic aneurysms. In an analysis of acute aortic dissection, a history of systolic blood pressure (SBP) of 180 mm Hg was cited as a risk factor for acute aortic dissection [15]. Perioperative blood pressure control is therefore of great importance for preventing intraoperative and postoperative complications. We previously reported that intraoperative blood pressure elevation can be controlled by administration of anxiolytics to patients with elevated blood pressure 2 hours before surgery [16].

In the present study, we focused on fluctuations in pulse rate (PR) 2 hours before surgery and investigated whether intraoperative blood pressure elevation could be better controlled by administration of anxiolytics to hosts with an increased PR.

## Patients and methods

### Ethics considerations

This clinical observational study was approved by the institutional review board of Miyata Eye Hospital (confirmation number CS-322) and complied with the tenets of the Declaration of Helsinki. Written informed consent was waived owing to the retrospective nature of the study, and patients were given the opportunity to withdraw from study participation via an opt-out procedure.

### Patients

This study included patients admitted to Miyata Eye Hospital (Miyazaki, Japan) who had undergone cataract surgery by phacoemulsification and intraocular lens insertion under local anesthesia. Patients were classified into 2 groups: in 1 group, early intervention for hypertension was performed on the basis of prior monitoring of changes in blood pressure and PR (PR group); in the other group, management was based on changes in blood pressure alone (conventional group). All patients in the PR group underwent cataract surgery between September and December 2018, whereas those in the conventional group underwent cataract surgery between September and December 2017. As previously reported [16], blood pressure was indirectly recorded at 5 time points: 2 hours before surgery, upon entrance into the operating room, at the beginning of surgery, at the end of surgery, and after surgery. We measured the PR before and after surgery and the heart rate upon the patient’s entrance into the operating room, at the beginning of surgery, and at the end of surgery using a biologic monitor in both groups in the same manner. To avoid confusion between heart rate and pulse rate in the text, we have unified the term to PR. Patients lacking blood pressure data at any time point, those with unstable pulse rate measurements due to arrhythmia, and those for whom follow-up could not be performed owing to insufficient medical information were excluded from the study.

### Circulatory management

Patients’ blood pressure and general condition were managed by a single physician following the standardized protocol based on the guidelines for the management of hypertension published by the Japanese Society of Hypertension [17]. In the conventional group, perioperative blood pressure control was initiated 2 hours before surgery, as previously reported [16]. The following SBP levels were considered hypertensive: >140 mm Hg before surgery, >160 mm Hg upon entrance into the operating room and at the beginning and end of surgery, and 140 mm Hg after surgery. Meanwhile, in the PR group, PR was defined as abnormal when elevated from the baseline (evaluated in the outpatient care unit before admission) by 10 beats/min. Additionally, the following SBP levels were considered hypertensive: >140 mm Hg before surgery, >180 mm Hg upon entrance into the operating room and at the beginning of surgery, >160 mm Hg at the end of surgery, and 140 mm Hg after surgery.

On the basis of the patient's clinical condition, nurses reported abnormal values to the physician. The attending physician initiated intravenous etizolam 0.5 mg (Towa Pharmaceutical) in the holding room before operation and nicardipine 0.5–1 mg (LTL Pharma) after entrance into the operating room.

### Preoperative blood pressure control

All patients underwent a preoperative examination by a physician. Regardless of the patients’ use of prescription medications for hypertension, those with a systolic/diastolic blood pressure of ≥130/85 mm Hg were defined as having hypertension in accordance with the relevant Japanese guidelines for hypertension [17]. Appropriate preoperative control of blood pressure was achieved using antihypertensive drugs such as angiotensin-converting enzyme inhibitors, angiotensin II receptor blockers, and calcium channel blockers, depending on the patient’s condition before surgery.

### Cataract surgery procedure

After hospitalization, cataract surgery was performed under local anesthesia with 4% xylocaine (Sandoz Pharmaceutical). Either corneal incision or sclerokeratotomy was performed, and 0.5 mL of 4% xylocaine was injected subconjunctivally in cases of sclerokeratotomy. After phacoemulsification and aspiration, the intraocular lens was fixed in the capsule. Finally, 0.3 mL dexamethasone (Sandoz Pharmaceutical), 1.5% levofloxacin (Santen Pharmaceutical), and ofloxacin ointment (Santen Pharmaceutical) were administered. After evaluation of vital signs, the patient was transferred to the ward and underwent examination by a physician, including assessments of blood pressure. Treatment with antihypertensive agents was continued in patients who still exhibited elevated SBP.

### Statistical analyses

The sample size was calculated before the planning of the study. For an effect size of 0.40 with 80% power and 5% alpha, at least 99 participants in each group were necessary to show a statistical significance. The chi-square test was performed to compare sex, history of hypertension, perioperative use of intravenous nicardipine and oral etizolam, and number of consultations with a physician. The Mann–Whitney U test was performed to compare patient age and operation time. Two-way ANOVA with the Sidak correction for multiple comparisons was performed to compare blood pressure, PR, and heart rate between the conventional and PR groups at each observation point. Data were expressed as means ± standard deviations; probability values of <.05 were considered significant.

## Results

In total, 684 eyes of 684 patients (aged 73.5 ± 9.5 years) were included in this study. The conventional and PR groups comprised 387 eyes of 387 patients and 297 eyes of 297 patients, respectively. No participants were excluded from the study. No significant differences in background characteristics such as age, male/female ratio, and operation time were observed between the 2 groups (Table 1).

**Table 1:** Patient characteristics

	Total	Conventional group	PR group	*P* value
*n*	684	387	297	–
	73.5 ± 9.5	73.0 ± 10.6	74.3 ± 8.5	.225
Male:female, *n*	268:416	162:225	106:191	.119
History of hypertension, n	382 (55.8%)	218 (56.3%)	164 (55.2%)	.832
Operation time, s	664.4 ± 392.6	668.3 ± 407.9	659.3 ± 372.0	.949

*PR* pulse rate

**Fig. 1:**
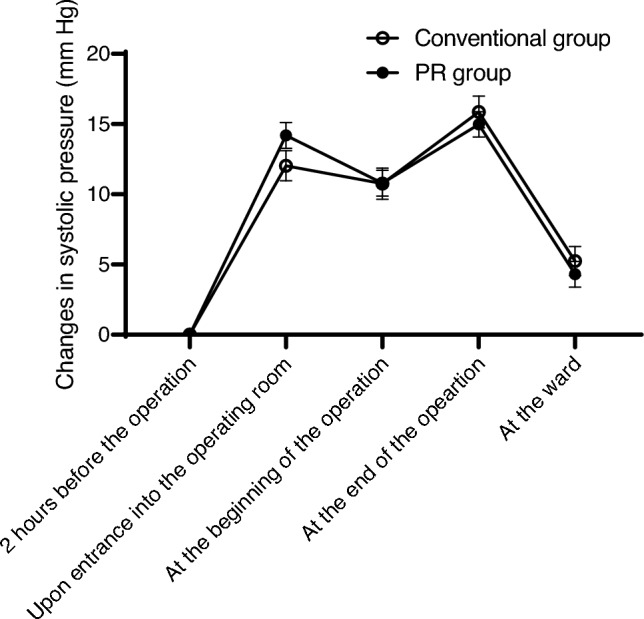
Changes in systolic blood pressure during the perioperative period. No significant differences in the changes in systolic blood pressure were found between the conventional and PR groups. *PR group* early monitoring/intervention group

**Fig. 2:**
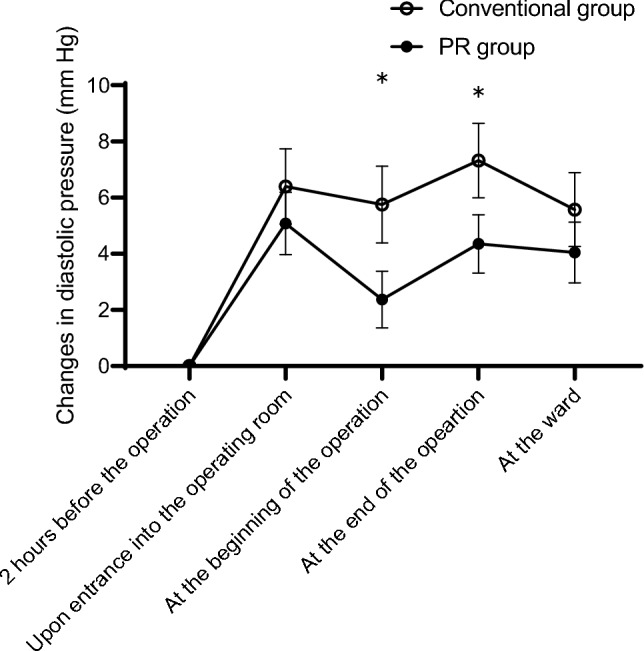
Changes in diastolic blood pressure during the perioperative period. Diastolic blood pressure was significantly lower in the PR group than in the conventional group at the beginning and end of the operation (*P* = .0005 and .0028). *PR group* early monitoring/intervention group

**Fig. 3:**
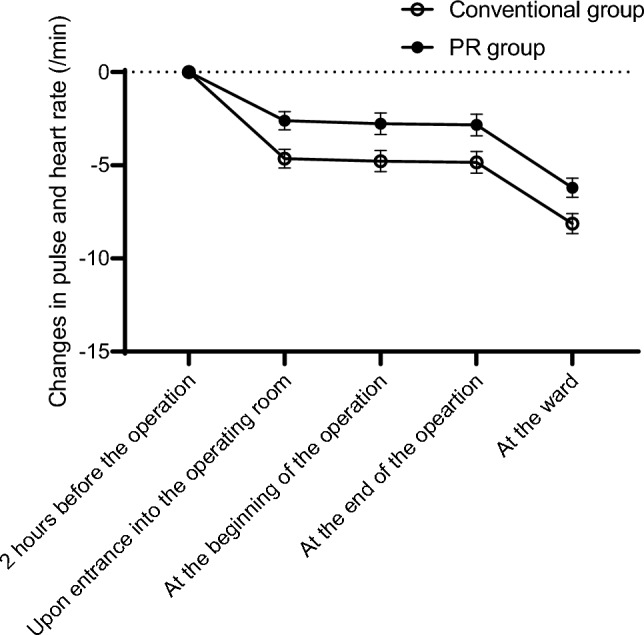
Changes in pulse and heart rates during the perioperative period. No significant differences in the changes in pulse and heart rates were found between the conventional and PR groups. *PR group* early monitoring/intervention gr

Changes in SBP from the baseline did not differ significantly between the conventional and PR groups at any observation point (Fig. 1). By contrast, changes in diastolic pressure from the baseline were significantly lower in the PR group than in the conventional group at the beginning and end of the operation (*P* <. 001 and *P =* .0028, respectively; Fig. 2). No significant differences were observed in pulse or heart rates between the groups (Fig. 3), and no serious general or ophthalmologic complications were observed in either group.

Intravenous nicardipine injection in the operating room was required significantly less in the PR group than in the conventional group (6.46% and 3.03%, *P* = .041). Furthermore, the PR group required significantly fewer nurse–physician consultations upon entering the operating room and at the beginning of the operation than did the conventional group (both *P* < .001; Table 2). Although perioperative oral etizolam usage significantly increased 2 hours before surgery (11.1% and 36.3%, *P* < .001), the usage upon entrance into the operating room significantly decreased in the PR group (15.5% and 7.4%, *P* < .001).

**Table 2:** Number of nurse–physician consultations during the perioperative period

	Total	Conventional group	PR group	*P* value
2 hours before surgery, n	200 (29.2%)	105 (27.1%)	95 (32.0%)	.167
Upon entrance into the operating room, *n*	79 (11.5%)	68 (17.6%)	11 (3.7%)	<.001
At the beginning of the operation, *n*	65 (9.5%)	53 (13.7%)	12 (4.0%)	<.001
At the end of the operation, *n*	152 (22.2%)	79 (20.4%)	73 (24.6%)	.194
In the ward, *n*	289 (42.3%)	153 (39.5%)	136 (45.8%)	.101

*PR* pulse rate

## Discussion

In this study, patients with an increased heart rate 2 hours before surgery were instructed to take antianxiety medication (as an increased heart rate is considered to reflect anxiety about surgery), and subsequent blood pressure changes were compared with those determined by conventional blood pressure changes alone. Cataract is a common disease in older adults, and many affected patients have comorbidities; untreated hypertension and diabetes are also often undetected. However, preoperative medical checkups are not required for minor noncardiovascular operations in Europe and the United States because they are quick and relatively physically noninvasive procedures. Host hypertension and sudden increases in blood pressure are recognized risk factors for the development of subarachnoid hemorrhage, aortic dissection, and aortic aneurysm rupture. Adequate management of diastolic blood pressure is clinically important in patients undergoing cataract surgery because postoperative elevation of blood pressure is associated with poorer general health and increase in the risk of abdominal aortic aneurysm rupture [18, 19]. In our previous study, early intervention 2 hours before surgery was more effective for controlling SBP than were conventional subjective methods, according to the physician’s judgement [16]. In the current study, we evaluated the effectiveness of early intervention on the basis of early monitoring of PR for the perioperative management of hypertension in patients undergoing cataract surgery under local anesthesia. Early detection of perioperative changes in blood pressure and PR after standardized intervention contributed to significantly decreased perioperative diastolic blood pressure during cataract surgery in the PR group when compared with the values observed in the conventional group. In addition, the need for intravenous nicardipine injection was significantly lower in the PR group than in the conventional group. In accordance with our previous findings, the current results demonstrate that early intervention based on PR reduced the need for nicardipine injections and aided in the control of diastolic blood pressure.

Meanwhile, overzealous attempts to reduce blood pressure may lead to an excessive postoperative drop in blood pressure, especially in patients with a history of hypertension [20]. Topical anesthesia results in a less dramatic decrease in diastolic blood pressure than does retrobulbar anesthesia [20]. For patients undergoing cataract surgery, peripheral vascular resistance may be elevated during the perioperative period owing to the beta effect of adrenaline, which is included in the medium used for phacoemulsification and aspiration. Additional studies are required to accurately assess the influence of these parameters on the basis of intraoperative measurements of catecholamine levels.

Perioperative anxiety is associated with an increase in pulse and heart rates during cataract surgery [1]. Although cataract surgery is relatively short in duration and is less invasive than ophthalmologic surgery of the posterior segment given the small size of the incision, its impact on the patient’s psychologic state cannot be ignored as 30% of patients experience emotional tension [21]. Preoperative anxiety can exert dramatic physiologic effects, leading to hypertension, tachycardia, hyperventilation, and hyperthermia. Preoperative anxiety has also been reported to prolong the phacoemulsification time, contributing to a longer surgical duration [22]. In the current study, although changes between the first and second surgeries were not evaluated, previous studies have reported lower levels of patient anxiety for follow-up surgeries [23]. Hand massage [24–26], music [27–29], and adequate preoperative education [30, 31] are effective in treating anxiety. A combination of these supportive therapies may be important for maintaining a consistent general health condition during the perioperative period.

Benzodiazepines are also effective in reducing anxiety [32]. To simplify care for patients undergoing cataract surgery under topical anesthesia without an anesthesiologist in the operating room, a previous group performed universal anxiolytic premedication using short-acting benzodiazepines (alprazolam 0.25 mg) provided at home on the day of surgery [33]. Therefore, we prefer to use preoperative sedatives after confirming any physiologic and psychologic changes. In the current study, we used 0.5 mg oral short-acting etizolam because it has a shorter half-life (5–7 h) than other short-acting benzodiazepines [34]. Essentially, our clinical research indirectly evaluated the ability to manage anxiety to aid in the management of hypertension. Since etizolam is an antipsychotic drug, it should be administered with caution to young patients or those with contraindications. In our hospital, etizolam is not used for young patients in principle. Notably, cataract patients under the age of 20 are very few in number, and furthermore, cataract surgery in anxious children is performed under general anesthesia because surgery under local anesthesia is difficult. Further, contraindications to etizolam include presence of primary/secondary angle closure or myasthenia gravis. Caution should be exercised in patients undergoing mydriasis for the first time during surgery owing to angle closure. Since the time to peak plasma concentration of etizolam is 0.5–2 hours, very early administration of this drug to patients with angle closure should be avoided [35].

Japanese Society of Hypertension guidelines for the management of hypertension state that surgery is the best opportunity to carry out an assessment of blood pressure and that if blood pressure is >180/110 mm Hg in a wait-listed procedure, doctors could consider postponing the operation [36]. Our analysis revealed no significant differences in age or history of hypertension between the 2 groups. Generally, older age is associated with greater arterial stiffness [37]. Accordingly, in patients with arteriosclerosis, higher peripheral arterial resistance is associated with higher diastolic blood pressure. By contrast, because diastolic blood pressure tends to be higher in younger patients with hypertension, the present protocol for managing PR and blood pressure may be particularly useful for younger patients with cataracts who may have comorbid diseases.

Interventions based on measurements of PR and blood pressure greatly reduce the time required for a physician to examine a patient. Intraoperative medical examinations are time-consuming and labor-intensive. Even when the consultation is performed in the operating room, such assessments prolong the operating time; increase the risk of infection at the surgical site; and increase the psychologic burden on the surgeon, medical staff, and patient. Therefore, reducing the operating room consultation time for cataract surgery is a valuable outcome for clinicians. Preoperative medical management is also essential for surgeries involving the use of local anesthesia, and several studies have demonstrated the improved safety and efficiency of preoperative risk-based medical evaluations [38]. Additionally, surgery may be postponed or abruptly cancelled for patients with poorly controlled medical conditions, leading to unnecessary health care costs and waste of medical resources. As such, medical conditions were adequately controlled in all the patients included in our study.

In our previous study, early intervention for blood pressure control 2 hours before surgery allowed us to maintain a lower perioperative SBP than would a blood pressure control method based on the subjective judgement of the surgeon [16]. The current study further demonstrated that interventions based on pulse control can lead to improved perioperative management of diastolic blood pressure elevation. Although the significant change in diastolic pressure was not high, the clinical outcomes, including fewer consultation times and reduced nicardipine usage, were effective for cataract patients. This advanced method may be useful in various clinical situations related to ophthalmic surgery, such as vitrectomy, eyelid surgery, and intravitreal injection, and it should be validated for such applications in future studies.

This study has several limitations. First, although our management protocol assessed increased pulse and heart rates, it did not involve the direct control of pulse and heart rates. We assumed that increased pulse and heart rates were a result of anxiety during the operations, as well as changes in blood pressure, and oral etizolam administration. Second, anxiety levels were not assessed in this study; therefore, our future study will focus on the preoperative monitoring of pulse and heart rates, which is easy to perform, related to preoperative anxiety during ophthalmic surgery.

In conclusion, the current results indicate that early assessment of PR and blood pressure and appropriate intervention thereafter can significantly reduce the requirement for intravenous nicardipine use and control the perioperative diastolic blood pressure values in patients undergoing cataract surgery under local anesthesia.

## Data Availability

Data supporting the results of this clinical research are available upon request to the corresponding author. Data are not available to the public because they contain information that may violate the privacy of the study participants.
